# Intestinal Biomarkers and Their Importance in Canine Enteropathies

**DOI:** 10.1155/vmi/7409482

**Published:** 2024-11-13

**Authors:** Iago Martins Oliveira, Rafaela Rodrigues Ribeiro, Maria Eduarda Cardoso Cysneiros, Larissa Barbosa Torres, Vanessa Rezende Moraes, Lucas Rodrigues Ferreira, Wanessa Patrícia Rodrigues da Silva, Murilo Rodrigues de Souza, Rafael Antônio Lopes Xavier, Paulo Renato dos Santos Costa, Danieli Brolo Martins, Naida Cristina Borges

**Affiliations:** ^1^Department of Veterinary Medicine, School of Veterinary and Zootechnics, Universidade Federal de Goiás, Rodovia Goiânia-Nova Veneza, km 8, Campus Samambaia, Goiânia 74690-900, Goiás, Brazil; ^2^School of Medical and Life Sciences, Pontifícia Universidade Católica de Goiás, Câmpus II, Av. Engler, s/n-Jardim Mariliza, Goiânia 74885-460, Goiás, Brazil; ^3^Veterinary Department, Universidade Federal de Viçosa, Avenida PH Rolfs, s/n University Campus, Viçosa 36570.900, Minas Gerais, Brazil

**Keywords:** biological indicators, canine, inflammation, intestine

## Abstract

Enteropathies are prevalent in dog internal medicine, and their diagnosis involves a lengthy process. One of the tests requested is for biomarkers, which are important as they can provide data on intestinal functionality, intensity of inflammation, and response to treatment, and can help determine the prognosis. This study aimed to conduct a literature review on the main serum and fecal intestinal biomarkers in dogs and proposed to refine the correlations between these indicators and enteropathies. It was observed that the main biomarkers used in the intestinal evaluation of dogs were alpha 1-proteinase inhibitory factor, immunoglobulin A, methylmalonic acid, serum folate, serum cobalamin, C-reactive protein, fecal and serum calprotectin, and dysbiosis index. However, we suggest that more research be carried out to clarify the relationship between enteropathies and intestinal biomarkers. We noticed a lack of studies on specific intestinal markers and indicator variables in healthy dogs and those with various enteropathies; moreover, no data are available on the association of these laboratory parameters.

## 1. Introduction

The intestines of dogs can be affected by diseases of various etiologies and are classified as chronic when there are clinical signs lasting longer than 3 weeks [1]. The most commonly described symptoms are vomiting, diarrhea, loss of appetite, and weight loss [2]. After ruling out infectious, neoplastic, metabolic, renal, and hepatic causes, intestinal disease can be divided into diet-responsive, antibiotic-responsive, immunosuppressant-responsive, and nonresponsive [3].

Serum and fecal biomarkers help diagnose and monitor the treatment of human intestinal diseases. Therefore, it is essential for the applicability of a biomarker to help assess the functionality of the organ, to recognize the risk of developing the disease, and to have diagnostic value [4]. However, due to the heterogeneity of canine enteropathies, a recent review [5] suggests new research into intestinal biomarkers for dogs.

Canine enteropathies can be expensive, time-consuming, and difficult to diagnose conclusively due to the nonspecificity of the clinical symptoms [5]. Thus, the emergence of new markers could help in the diagnosis of intestinal diseases. In human medicine, serum and fecal biomarkers have been used in inflammatory bowel disease for many years [7]. Some of the most commonly used indicators are hematometric parameters such as leukometry and platelet assessment as well as the measurement of C-reactive protein. The most commonly studied fecal markers are lactoferrin and fecal calprotectin. However, although these are routine tests, they are not specific to enteritis [8].

In canine intestinal diseases, these markers can be classified as functional, biochemical, inflammatory, cellular, genomic, proteinomic, metabolomics, and microbiomic [9]. Based on the number of cases, costs, and information on the methods available for evaluating the intestines of dogs, the most widely available markers in clinical studies for diagnosing and monitoring diseases are functional and inflammatory [6].

Therefore, to the best of our knowledge, there is still a gap in the applicability of tests for gastrointestinal evaluation in animals. The aim of this literature review was to address the intestinal evaluation of dogs using these laboratory parameters as well as the importance of indicators in enteropathies.

## 2. Laboratory Criteria for Evaluating a Biomarker

When interpreting laboratory tests, it must be considered whether the analyte is qualitative (positive or negative) or quantitative, where the result is ordinal, usually numerical, based on reference values [10]. Therefore, it is crucial to know the characteristics of each test. Furthermore, interpretation should be done while considering information from the anamnesis, physical examination findings, and other clinical, laboratory, and imaging tests [9, 11].

Preanalytical factors are essential for the quality of tests as well as for diagnostic performance, as the collection of biological material, transportation, and storage influence the results. The professional's technical skills, the method used to perform the tests, and the instruments used can also affect the analysis [12].

As analytes can show individual and interpopulation biological variability, this aspect needs to be considered when interpreting marker findings [13]. In intestinal function, variation can occur due to gastrointestinal transit, differences in secretions present in the lumen, cellular distribution in which measurement occurs at fecal level, and half-life of the analyte. Thus, strategies are carried out to minimize these influences, such as serial collection or specific laboratory analysis methodology [6, 13].

Furthermore, the diagnostic accuracy of a biomarker is described by its specificity and sensitivity [9]. Sensitivity is the probability of the result being positive in the sick individual and is calculated by the ratio between those positive for the disease and the total population of sick individuals. Consequently, the higher the sensitivity is, the lower the false negative rate is. Therefore, biomarkers are useful for screening and ruling out diseases. Thus, specificity is the probability of the result being negative in the nonsick individual and is calculated by the ratio between those negative for the disease and the total population of nonsick individuals. Thus, the higher the specificity is, the lower the false positive rate is. Consequently, confirmatory tests are important [14, 15].

Accuracy of an analyte is related to its diagnostic performance and can be determined using predictive values [9, 14]. These are important in terms of clinical applicability as a biomarker for an organ, especially because they help to select the patient profile in which the test is likely to produce relevant results [10]. The positive predictive factor is the probability of the patient being positive and having the disease; thus, these tests are most commonly applied to individuals with a high suspicion of the disease and without interferences such as medication and comorbidities. Conversely, the negative predictive value is the probability that the negative patient does not actually have the disease; thus, it can be considered in the evaluation of large populations and is contraindicated in specific suspicions of a condition [15].

The biomarkers for intestinal assessment in dogs are summarized in Table 1. Based on the information obtained in this literature review, we believe that the low number of intestinal markers used in dogs may be related to the cost of performing the tests, issues related to standardization among veterinary laboratories, and lack of technical information on the methodology used to perform the tests.

Heilmann and Steiner [6] suggest that the goals of using intestinal biomarkers are to assess the risk of developing diseases, provide a diagnosis, reflect on intestinal function, determine the severity of the disease, evaluate responses to treatment, monitor the disease, and detect relapses in chronic cases.  Table 2 presents a compilation of studies on the main serum and fecal intestinal biomarkers in dogs.

## 3. Functional Intestinal Biomarkers

### 3.1. Alpha 1-Proteinase Inhibitor

Alpha 1-proteinase inhibitor (*α*1PI) is an inhibitory factor for serine proteases, which are enzymes that cleave proteins. It protects the body by neutralizing various proteolytic enzymes. It is mainly synthesized by hepatocytes but can be produced by other cells, such as enterocytes [16]. Hepatic synthesis occurs continuously, and there is no deposition in this organ. Impairment of the activity of this marker would therefore lead to poor protection from the action of macrophage proteases as well as reducing the capacity of the response to systemic inflammation [17].

It has a similar molecular weight to albumin and in diseases where there is intestinal protein loss, and both can be reduced in serum [18]. However, unlike albumin, *α*1PI is resistant to proteolysis, which allows for fecal assessment. Despite this, the contribution of this factor to inflammation is still uncertain [19].

**Figure 1:**
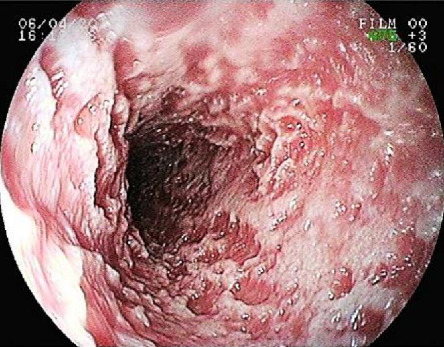
Upper digestive endoscopy of a dog with primary intestinal lymphangiectasia. Evaluation of the duodenum revealed an irregular whitish surface, suggesting significant lymphatic ectasia, hyperemia, and edema of the mucosa.

In human medicine, this factor acts as a negative acute phase protein [20]. Regardless of this, in dogs, there is only a suggestion that it acts in inflammatory processes, and these studies in veterinary medicine favor the species-specific role of *α*1PI in the pathophysiology of diseases [21, 22]. There have been reports of a reduction in serum levels of this marker in cases of systemic inflammation, sepsis, pancreatitis, and exocrine pancreatic insufficiency [23–25]. Protein-losing enteropathy can cause a reduction in the concentration of this biomarker [26, 27].  Figure 1 illustrates an endoscopic image of a dog with intestinal lymphangiectasia, which normally shows protein loss and, consequently, a reduction in absorption markers such as *α*1PI.

Increased fecal counts in dogs suggest impaired intestinal absorption and protein loss, as seen in intestinal lymphangiectasia and intestinal crypt abscesses [28]. As there is variation in fecal elimination, it is suggested that feces be collected on three consecutive days, for measurement. This biomarker is considered for early detection of protein loss from the gastrointestinal tract, since its fecal increase occurs before hypoalbuminemia and the manifestation of intestinal protein loss [29]. It also helps to differentiate intestinal protein loss from liver failure and kidney disease with proteinuria [28, 29].

Secondary to gastrointestinal loss through feces, there is a reduction in serum *α*1PI, and the serum and fecal ratio is a method of aiding in the diagnosis of protein-losing enteropathies in dogs [28].

### 3.2. Immunoglobulin A

Immunoglobulin A (IgA) is predominantly observed in secretions and is associated with mucosal immunity. Thus, measurement of fecal IgA can act as a biomarker of gastrointestinal immunity, as its value in the feces may demonstrate the synthesis and secretion of this immunoglobulin by the intestine [30]. A study in dogs sought to prove that fecal IgA was related to intestinal immunity [31]. In German Shepherds, it was experimentally confirmed that there is a deficiency of fecal IgA in enteropathies, and it was suggested that this reduction was associated with the functional incompetence of intestinal plasma cells due to the low constancy of IgA concentration in the feces. However, this study did not conclude whether this change was a cause or a consequence of the intestinal disease [31, 32].

To assess the value of fecal IgA, it is necessary to consider physiological characteristics such as breed, age, and body condition score as well as factors associated with the feces themselves, such as moisture and quantity. As there is a variability in dog breeds, physical size can influence the diameter of the gastrointestinal tract, which is correlated with fecal passage rate, stool moisture, and volume [33]. Based on this, a study evaluated whether IgA is influenced by stool moisture and whether this marker can be useful in the diagnosis of intestinal disorders caused by different enteropathogens with slopes based on the breed and size of the dog. It has been found that IgA has no diagnostic value for detecting intestinal pathogens in healthy dogs and dogs with diarrhea, but it helps to understand the development of the intestine with age [34].

Although a reduction in the fecal concentration of IgA can be seen in dogs with chronic intestinal disorders, the information currently available in the literature does not provide sufficient support for the clinical application of this measurement in dogs with gastrointestinal clinical signs [6].

### 3.3. Cobalamin and Folate

**Figure 2:**
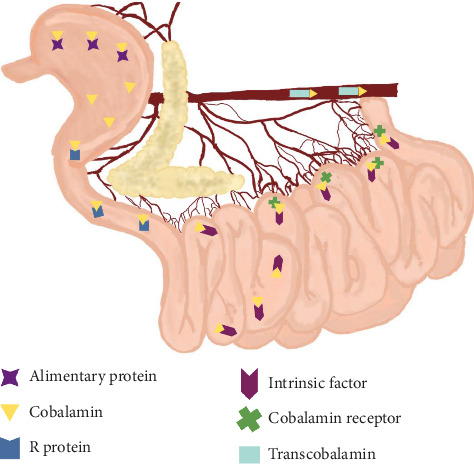
Schematic of cobalamin absorption in dogs. Food protein is cleaved in the stomach to produce cobalamin. It is initially transported by the R protein and later by the intrinsic factor. Absorption takes place in the distal small intestine (ileum). The stomach, enzymes, pancreas, receptors, and specific transporters all play an important role. Figure created with Penup Samsung application (Windows 11, Ldta) and Canva Pro.

Cobalamin is also known as cyanocobalamin or vitamin B12. It is part of the B-complex vitamins, is water-soluble, and acts as a cofactor for intracellular enzymatic reactions [35]. B12 obtained from the diet is cleaved and released in the stomach by the action of gastric acid and is subsequently associated with gastric protein R, which generates a transport complex to the duodenum [35]. In this region, the complex is disintegrated by pancreatic proteases and cobalamin is conjugated to the intrinsic factor [36]. The product of this conjugation is absorbed only in the ileum. After absorption, cobalamin is distributed to the tissues associated with another carrier protein, transcobalamin [37] (Figure 2).

Production of intrinsic factor is species-specific. In humans, it is produced by the parietal cells of the gastric mucosa, and in dogs, it is produced in the stomach and by the exocrine pancreas [36], whereas in cats, the synthesis of this glycoprotein is considered to be exclusive to the exocrine pancreas [38]. Given that the main source of this glycoprotein in dogs is the pancreas, a common etiology of hypocobalaminemia in this species is exocrine pancreatic insufficiency [36].

As B12 is absorbed in the distal small intestine, ileitis can cause malabsorption of this vitamin due to reduced expression of the receptor, inflammation of the mucosa, or impaired expression of the intrinsic factor receptor. Thus, cobalamin deficiency is commonly observed in dogs with chronic enteropathy [39]. Additionally, intestinal dysbiosis leads to hypocobalaminemia through increased bacterial consumption and microbial competition for dietary B12 [35, 40]. Deficiency of this vitamin has been described in cases of hereditary predisposition, associated with the racial pattern of dogs, selective malabsorption, and intestinal diseases, and it is also known to occur in alimentary lymphoma [40]. It is associated with an unfavorable prognosis and is related to hypoalbuminemia in severe cases of intestinal disease [41].

Cobalamin is a nonspecific marker, and normal levels cannot rule out intestinal disease. Serum levels below the reference level require parenteral or oral supplementation, since, clinically, dogs with this vitamin deficiency can present lethargy, loss of appetite, vomiting, diarrhea, weight loss, and growth retardation [42, 43].

Hypercobalaminemia in humans is associated with hematological abnormalities and neoplasms [44], but in animals, there are few studies on the clinical impact that can be caused by excess serum vitamin B12 [40]. Previously, this excess serum vitamin B12 was considered benign and of no clinical relevance to small animals [6]. However, a recent study aimed to investigate the association of hypercobalaminemia with neoplastic processes in dogs and cats as well as to determine the clinical and pathological conditions associated with this change. The results suggested no correlation between neoplasms and oncogenesis and cobalamin, but there was an association with inflammatory diseases such as pancreatitis [45].

**Figure 3:**
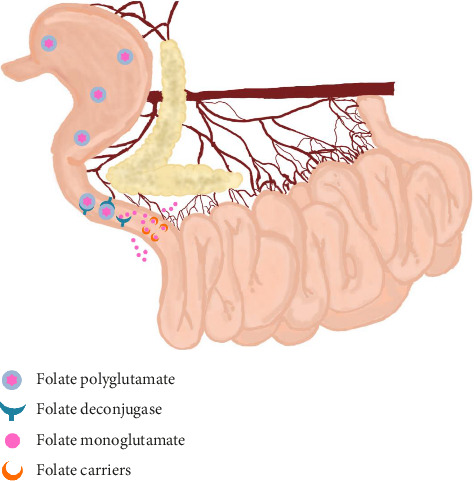
Schematic of folate absorption in dogs. Folate polyglutamate from the diet is cleaved by folate deconjugase, which gives rise to folate monoglutamate. This, in turn, is transported by specific carriers to the proximal small intestine (duodenum and jejunum) where absorption takes place. Specific enzymes, receptors, and transporters play an important role. Figure created with Penup Samsung application (Windows 11, Ldta) and Canva Pro.

Folate, also known as folic acid or vitamin B9, has both plant and bacterial origins [46]. It is a water-soluble vitamin absorbed in the duodenum, particularly in the jejunum, in the form of folate monoglutamate via specific transporters (Figure 3). The reduction of serum folate can occur due to malabsorption in the proximal intestine that occurs in inflammatory processes, but a reduction in serum B9 or normofolatemia cannot rule out enteropathy in dogs, and is therefore a nonspecific intestinal biomarker [47]. Hyperfolatemia can occur in cases of dysbiosis due to the production of folate by some intestinal microorganisms, and in these cases, there is usually an association with hypocobalaminemia [48].

Furthermore, in human medicine, an increase in serum folate is observed in cases of hemolysis, but there is little evidence of this change in veterinary medicine [49, 50]. Recently, in contrast to studies in humans, a study in dogs revealed a low correlation between hemolysis and a reduction in B9 in the blood. Furthermore, this study concluded that folate is an intestinal biomarker, but that it is inferior to cobalamin for the prognosis of chronic canine enteropathies and that hypopholatemia is not associated with findings of severity such as hypoalbuminemia and more pronounced clinical changes [50].

Thus, hypocobalaminemia and hypopholatemia suggest intestinal malabsorption and help locate the lesion in the ileum or duodenum/jejunum regions where B12 and B9 are absorbed, respectively [6]. A deficiency of these vitamins can compromise the synthesis of deoxyribonucleic acid (DNA), which interferes with hematopoiesis and cell replication. Despite this, it was noted that macrocytosis and regenerative anemia are not common in dogs with B12 and B9 deficiency, as is observed in humans [51].

### 3.4. Methylmalonic Acid (MMA)

Methylmalonyl-coenzyme A (CoA) mutase catalyzes the formation of succinyl-CoA from methylmalonyl-CoA, and the product of this enzymatic reaction is important for the essential composition of the tricarboxylic cycle. Since this converting enzyme depends on the action of cobalamin, intracellular deficiency of this vitamin leads to a reduction in enzyme activity and accumulation of intracellular MMA [52, 53].

Increase in MMA production is useful in identifying intracellular B12 deficiency as a possible consequence of impaired absorption, competition for uptake, decreased cobalamin transport, or a combination of these possibilities. This biomarker can be measured in urine or serum. The interpretation of the amount of serum cobalamin associated with serum or urinary MMA is superior to the evaluation of serum B12 alone in dogs [26, 54].

Measurement is not routinely performed on animals because it is tedious to perform and expensive [55]. It is suggested that measuring serum MMA in dogs is a more sensitive biomarker for assessing cobalamin metabolism in the body, since serum B12 may not express cellular concentrations [35]. Serum MMA levels in dogs can increase owing to conditions not related to impaired intestinal absorption or dysbiosis, such as in chronic kidney disease; thus, to aid interpretation, it is suggested that renal markers be requested, such as serum creatinine and symmetrical D-methyl-arginine [54].

## 4. Microbiological Intestinal Biomarkers

### 4.1. Coproculture

Stool culture is a diagnostic tool used to identify specific enterobacteria such as *Salmonella* spp., *Campylobacter jejuni*, specific enteropathogenic strains of *Escherichia coli*, *Yersinia* spp., *Clostridium perfringens*, *Clostridium difficile,* and fungi in animals with diarrhea [56].

This test has numerous limitations for determining the etiology of diarrhea due to the lack of standardization of the techniques carried out in different laboratories, such as the quantity of feces sent, packaging for sending (frozen, refrigerated, or room temperature), and the methodology of execution in the types of culture medium and dilution of sampling [57]. Additionally, most intestinal bacteria, especially those that colonize the large intestine, are strict anaerobes, which require specific culture media [58].

Coprocultures carried out in veterinary diagnostic laboratories may underestimate the amount of bacteria present in feces due to the use of common culture media or because they have limited anaerobic culture media [59]. Therefore, culture is not indicated as a diagnostic method to determine whether the feces are normal or abnormal in terms of microbial load, especially in dogs with chronic diarrhea [57]. Furthermore, clinical usefulness of the test is questionable since healthy patients have enteropathogens isolated in their feces, and these agents may not be isolated in dogs with diarrhea [59].

Recently, in a prospective case–control study, fecal samples sent to different laboratories were analyzed for fecal culture and fecal molecular evaluation using polymerase chain reaction (PCR). In this study, it was noted that coproculture does not allow us to distinguish between sick and healthy animals. Additionally, there was a lot of variability in the results from different laboratories with the same samples [59].

### 4.2. Dysbiosis Index (DI)

DI is considered a microbiological fecal biomarker because it indicates changes in the intestinal bacterial microbiome. It assesses the normobiosis and dysbiosis profile by identifying seven groups of bacteria that are normally altered in dogs with enteropathy, more specifically, those responsive to immunosuppressants: *Blautia* spp, *Clostridium hiranonis*, *Escherichia coli*, *Faecalibacterium* spp., *Fusobacterium* spp., *Streptococcus* spp., and *Turicibacter* spp., and total bacteria, with 74% sensitivity and 95% specificity [60, 61] (Table 3).

This index is carried out using quantitative PCR. In addition to promoting intestinal microbial measurement with individual and specific results for groups of bacteria, it combines these groups using a logarithmic mathematical algorithm [64, 65]. PCR is a useful method for quantifying the intestinal microbial load of dogs and is considered a rapid test with good reproducibility in identifying these clinically relevant microorganisms [61, 63].

The ID has a reference range of 0–2; values above the reference are considered to be dysbiosis, and the higher the ID value, the greater the discrepancy with the normal gastrointestinal microbiota. In intestinal dysbiosis, there are various changes, including a reduction in the production of *Clostridium hiranonis* and an increase in *Streptococcus* spp. and E. *coli* [64]. The reduction in C. *hiranonis* is related to a change in the conversion of primary bile acids into secondary bile acids [65]. With this change, the physiological control of intestinal bacteria is compromised, and pathogens such as *Clostridium difficile*, *E. coli*, and *Clostridium perfringens* proliferate [66]. ID can therefore be used in cases of dysbiosis associated with enteropathies as well as for understanding the repercussions of antibiotic use and for evaluating fecal microbiota transplantation [59, 67].

## 5. Inflammatory Intestinal Biomarkers

### 5.1. C-Reactive Protein

C-reactive protein is classified as a positive acute phase protein, acting as a marker of the inflammatory response to various inflammatory, infectious, and neoplastic processes [68, 69]. It is synthesized in the liver and is composed of five subunits. Its serum level increases secondary to pro-inflammatory cytokines, such as interleukin (IL) 1, 6, and tumor necrosis factor alpha, and this increase is proportional; thus, the higher the inflammation and release of cytokines is, the higher the level of this test is [70, 71]. Elimination occurs through the liver as the underlying cause is resolved, so it can be used clinically as an inflammatory biomarker [71].

It is a nonspecific protein towing to the variability of etiologies that can cause its serum increase, which makes it difficult to use as a diagnostic biomarker in dogs with chronic enteropathy; however, it is used as a marker to assess disease progression, responses to treatment, and prognosis [72–74]. For the change to be considered relevant in clinical medicine, the values need to be 2.7 times higher than the maximum limit of the reference interval [75].

In gastroenterology, this biomarker can be useful not only in monitoring therapeutic response and prognosis but also in indicating treatments. An example of this is a study which found that dogs with C-reactive protein above 9.1 mg/L needed to be treated with immunosuppressive drugs, while dogs with lower serum values were treated with diet or antibiotics, with a sensitivity of 72% and specificity of 100% [73].

C-reactive protein as a biomarker was used in a prospective study of dogs with chronic intestinal inflammation treated with immunosuppressants. A reduction in the serum concentration of this protein was observed in the treated animals when compared to those in the control group [76]. In another similar study, the value was significantly higher in animals with intestinal disease at the time of diagnosis when compared to healthy dogs [77].

Elevations in serum C-reactive protein level should not be considered etiological diagnostic findings, due to their nonspecificity. However, it is highly sensitive and has the potential to provide diagnostic support for animals with subclinical diseases [77]. A recent review on C-reactive protein applications in gastrointestinal diseases in dogs concluded that measurement in veterinary medicine, based on results from its use in human gastroenterology, is promising and tends to be used in new therapeutic approaches [78].

### 5.2. Calprotectin

Calprotectin is the name of the heterodimer of S100A8/S100A9. It binds to calcium and is associated with inflammatory processes, especially chronic ones [80]. It is a molecular pattern associated with damage of the innate immune response expressed by activated macrophages, neutrophils, and epithelial cells that bind to toll-like receptors, which, in turn, are transmembrane proteins associated with signal formation and production of inflammatory mediators of innate immunity [81, 82]. Additionally, these proteins collaborate in the regulation of cell proliferation and act as intracellular damage signalers. Hence, they are known as alarmins [9].

Therefore, fecal calprotectin is a low-invasive, accessible biomarker that can be used for the diagnosis, classification, response to treatment, and prognosis of dogs with chronic enteropathy. The fecal measurement of this marker is more sensitive and specific than the serum value in dogs with diseases of the gastrointestinal tract. This is because the serum level of calprotectin can increase due to extra-intestinal diseases, and its value may be influenced by drugs, such as corticosteroids [83].

Calprotectin can determine the severity of intestinal disorders in dogs. Moreover, it is useful in understanding the response to treatment in canine patients, as it was observed that lower values were detected in the feces of animals that responded fully or partially to treatment, to the detriment of those that had higher fecal values and an inadequate response or complete irresponsiveness to therapy, with 80% sensitivity and 70% specificity. There are also studies that prove the relationship between this biomarker and the severity of clinical signs and histopathological lesions [84]. However, there are still no studies evaluating the effects of gastrointestinal neoplasms on fecal calprotectin values [6].

### 5.3. Calgranulin C

Calgranulin C is a heterodimer of the S100A12 protein complex and acts as a molecular pattern associated with damage to various target proteins, specifically the receptor for advanced glycation products that modify the inflammatory and immunological cascades [85]. It is synthesized by activated mononuclear cells, has fecal stability which allows it to be measured, and is sensitive and specific for localized inflammatory processes [86].

Severity of clinical symptoms and endoscopic findings are correlated with the fecal value of calgranulin C, but studies have not yet shown a relationship with histology [85]. It can also be used to monitor the treatment of dogs with intestinal disease as well as to classify animals with this condition based on the responsiveness of the therapy and severity of the case [87].

It was noted that fecal elevations of this analyte can distinguish animals that need immunosuppressive medication from those that can be treated with an exclusion diet, hydrolyzed protein, or antibiotics, with 64% sensitivity and 77% specificity. As with calprotectin, there are no studies evaluating the effects of gastrointestinal neoplasms on fecal calgranulin C values [6].

### 5.4. Other Inflammatory Intestinal Biomarkers

3-Bromotyrosine is a metabolic product of enzymes released by eosinophils after activation and respective degranulation and, therefore, is a biomarker of eosinophilic inflammation [87, 88]. Therefore, this marker may be present in dogs with enteritis, which, despite more commonly presenting a lymphoplasmacytic infiltrate in the lamina propria of the mucosa, may also have an isolated or mixed eosinophilic infiltrate [89].

Studies reveal that the fecal and serum concentration of this marker may be increased in dogs with gastrointestinal disorders, but there is still no standardization or studies to determine specificity and sensitivity [6]. A study was recently carried out to evaluate the diagnostic sensitivity of serum and fecal measurements of 3-bromotyrosine in dogs through clinical and histopathological comparisons. In that study, it was found that the marker increased in the serum of animals with chronic intestinal disease, but that there was no relationship with clinical symptoms or histological lesions [90].

Several studies suggest the use of pro-inflammatory cytokines, such as ILs, to assess inflammation in intestinal diseases: IL-2, IL-4, IL-5, IL-6, IL-8, IL-10, IL-12, IL17, IL-18, IL-23, IL-25, and IL-33 [91]. However, the use of these molecules as intestinal laboratory parameters is limited, as there are many clinical trials available without specific validation [6]. Thus, for canines, combined measurement and correlation with other biomarkers is suggested.

## 6. Conclusion

Owing to the anatomical and functional complexity of the gastrointestinal tract of dogs, frequent diseases that affect the intestines of these animals, and the presence of nonspecific clinical signs, auxiliary diagnostic methods are crucial. Among these, biomarkers play a significant role.

An analysis of the descriptions of the sources used here shows that there is a wide variety of markers, mainly serum and fecal, and that these have potential for assessing intestinal health and for diagnosing, prognosing, and monitoring enteropathies. These factors highlight the growing importance of combining different complementary tests to understand the pathophysiology of diseases affecting the canine digestive system.

Notably, there is still a need for studies on the methodology for applying biomarkers to standardize reference measurements and practical clinical application as well as the need to discover specific markers for intestinal assessment in dogs. As research on canine intestinal function advances, the need for studies with larger sample sizes and control tests with healthy dogs and dogs with different enteropathies is warranted.

## Figures and Tables

**Table 1:** Main intestinal biomarkers in dogs and their classification according to the type of evaluation.

Intestinal biomarkers	
Functional biomarkers	Serum alpha 1-proteinase inhibitor
	Fecal alpha 1-proteinase inhibitor
	Serum folate
	Serum cobalamin
	Methylmalonic acid
	Fecal immunoglobulin A

Inflammatory biomarkers	C-reactive protein
	Perinuclear antineutrophil cytoplasmic antibody
	Fecal calprotectin
	Serum calprotectin
	Fecal calgranulin
	Serum calgranulin
	Serum bromotyrosine
	Serum *N*-methylhistamine
	Urinary *N*-methylhistamine
	Inflammatory cytokines
	Inflammatory chemokines
	Fecal alkaline phosphatase

Genomic biomarkers	Single-nucleotide polymorphism
	Gene expression changes

Reference	[9]

**Table 2:** Serum intestinal biomarkers characterized in terms of biological domain, methodology, and reference values for dogs.

Biomarker	Biological field	Methodology	Reference values for dogs	Reference
Fecal *α*1PI	Protein	High-performance liquid chromatography	≥ 19.0 μg/g	[28]
Serum *α*1PI	Protein	High-performance liquid chromatography	≤ 1.087 mg/L	[28]
Fecal/serum *α*1PI ratio	—	—	≤ 53.6 g/mL	[28]
Fecal immunoglobulin A	Immunoglobulin	Enzyme-linked immunosorbent assay	≥ 0.22 mg/g	[32]
Serum cobalamin	Vitamin	Electrochemiluminescence	400–908 ng/L	[41]
Serum folate	Vitamin	Electrochemiluminescence	3.5–8.5 ng/mL	[49]
Serum methylmalonic acid	Amino acid	Gas chromatography	415–1193 nmol/L	[56]
Serum C-reactive protein	Protein	Immunochromatography	≤ 0–8.0 mg/L	[75]
Dysbiosis index	—	PCR	0–2^a^	[61]
Fecal calprotectin	Protein	Immunochromatography	≥ 15.2 μg/g	[84]
Serum calprotectin	Protein	Immunochromatography	296 μg/L	[84]
Fecal calgranulin	Protein	Immunochromatography	273 ng/g	[84]
Serum calgranulin	Protein	Immunochromatography	No reference value	[9]

Abbreviations: α1PI, alpha 1-proteinase inhibitor; PCR, polymerase chain reaction.

^a^Unit of measurement is expressed as log DNA bacteria/gram of feces; μg/g, microgram per gram; mg/L, milligram per liter; g/mL, gram per liter; mg/g, milligram per gram; ng/L, nanogram per liter; ng/mL, nanogram per milliliter; nmol/L, nanomol per liter; g/L gram per liter; and ng/g, nanogram per gram.

**Table 3:** Dysbiosis index in dogs.

Bacteria	Function	Normal value for dogs^a^	Change in dysbiosis
*Faecalibacterium*	Anti-inflammatory and production of SCFA	3.4–8.0	Reduction
*Turicibacter*	SCFA production	4.6–8.1	Reduction
*Blautia*	SCFA production	9.5–11.0	Reduction
*Fusobacterium*	SCFA production	7.0–10.3	Reduction
*Bifidobacterium*	SCFA production	Not measured	Reduction
*Bacteroides*	SCFA production	Not measured	Reduction
*Clostridium hiranonis*	Conversion of primary bile acids into secondary bile acids	5.1–7.1	Reduction
*Streptococcus*	Proliferation associated with dysbiosis	1.9–8.0	Increase
*Escherichia coli*	Pro-inflammatory	0.9–8.0	Increase
Dysbiosis indexa	< 0: Normal dysbiosis index, which suggests that there are no changes in the overall diversity of the intestinal microbiota.		
	0–2: Mildly increased dysbiosis index, suggesting a mild to moderate change in the overall diversity of the intestinal microbiota.		
	> 2: Dysbiosis index significantly, significantly, suggesting a marked change in the overall diversity of the intestinal microbiota.		
	[60]		

*Note:* Bacterial agents evaluated with their activity in the intestinal microbiome, change in microbiota in intestinal dysbiosis, reference values for dogs, and numerical value of the extent of intestinal dysbiosis in dogs.

Abbreviation: SCFA, short-chain fatty acid.

^a^Unit of measurement is expressed as log DNA bacteria/gram of feces.

## Data Availability

Data sharing is not applicable to this article as no datasets were generated or analyzed during the current study.

## Nomenclature

α1PI: Alpha 1-proteinase
DNA: Deoxyribonucleic acid
ID: Dysbiosis index
IgA: Immunoglobulin A
IL: Interleukin
MMA: Methylmalonic acid
PCR: Polymerase chain reaction
